# What does heritability of Alzheimer’s disease represent?

**DOI:** 10.1371/journal.pone.0281440

**Published:** 2023-04-28

**Authors:** Emily Baker, Ganna Leonenko, Karl Michael Schmidt, Matthew Hill, Amanda J. Myers, Maryam Shoai, Itziar de Rojas, Niccoló Tesi, Henne Holstege, Wiesje M. van der Flier, Yolande A. L. Pijnenburg, Agustin Ruiz, John Hardy, Sven van der Lee, Valentina Escott-Price

**Affiliations:** 1 Division of Neuroscience and Mental Health, School of Medicine, Cardiff University, Cardiff, United Kingdom; 2 Dementia Research Institute, School of Medicine, Cardiff University, Cardiff, United Kingdom; 3 School of Mathematics, Cardiff University, Cardiff, United Kingdom; 4 Department of Cell Biology, Miller School of Medicine, University of Miami, Coral Gables, FL, United States of America; 5 Institute of Neurology, University College London, London, United Kingdom; 6 Research Center and Memory Clinic, ACE Alzheimer Center Barcelona, Universitat Internacional de Catalunya, Barcelona, Spain; 7 Networking Research Center on Neurodegenerative Diseases (CIBERNED), Instituto de Salud Carlos III, Madrid, Spain; 8 Genomics of Neurodegenerative Diseases and Aging, Human Genetics, Vrije Universiteit Amsterdam, Amsterdam UMC location VUmc, Amsterdam, The Netherlands; 9 Amsterdam Neuroscience, Neurodegeneration, Amsterdam, The Netherlands; 10 Delft Bioinformatics Lab, Delft University of Technology, Delft, The Netherlands; 11 Alzheimer Center Amsterdam, Neurology, Vrije Universiteit Amsterdam, Amsterdam UMC location VUmc, Amsterdam, The Netherlands; Forest Research, UNITED KINGDOM

## Abstract

**Introduction:**

Both late-onset Alzheimer’s disease (AD) and ageing have a strong genetic component. In each case, many associated variants have been discovered, but how much missing heritability remains to be discovered is debated. Variability in the estimation of SNP-based heritability could explain the differences in reported heritability.

**Methods:**

We compute heritability in five large independent cohorts (N = 7,396, 1,566, 803, 12,528 and 3,963) to determine whether a consensus for the AD heritability estimate can be reached. These cohorts vary by sample size, age of cases and controls and phenotype definition. We compute heritability a) for all SNPs, b) excluding *APOE* region, c) excluding both *APOE* and genome-wide association study hit regions, and d) SNPs overlapping a microglia gene-set.

**Results:**

SNP-based heritability of late onset Alzheimer’s disease is between 38 and 66% when age and genetic disease architecture are correctly accounted for. The heritability estimates decrease by 12% [SD = 8%] on average when the *APOE* region is excluded and an additional 1% [SD = 3%] when genome-wide significant regions were removed. A microglia gene-set explains 69–84% of our estimates of SNP-based heritability using only 3% of total SNPs in all cohorts.

**Conclusion:**

The heritability of neurodegenerative disorders cannot be represented as a single number, because it is dependent on the ages of cases and controls. Genome-wide association studies pick up a large proportion of total AD heritability when age and genetic architecture are correctly accounted for. Around 13% of SNP-based heritability can be explained by known genetic loci and the remaining heritability likely resides around microglial related genes.

## 1. Introduction

Autosomal dominant Alzheimer’s disease accounts for only ~1% of all cases, the remaining AD cases are probably caused by a complex interplay of environmental and genetic factors. The pathological changes of aggregation of amyloid plaques and formation of intracellular neurofibrillary tangles begin in the brain long before manifestation of the first clinical symptoms due to severe neuronal loss [1]. AD can be diagnosed with certainty during life using cerebrospinal fluid (CSF) biomarkers, amyloid PET imaging and definitely at autopsy [2, 3]. However, the accuracy of clinical diagnosis, without the use of CSF or blood biomarkers or PET imaging, is relatively low and includes up to 30% of misdiagnosed patients [4–6].

The heritability (the proportion of phenotypic variance explained by genetics [7]) of late onset Alzheimer’s Disease liability is generally agreed to be between 60–80% from twin studies [8, 9]. The largest contributor to genetic risk is the *APOE* gene and genome-wide association studies (GWAS) have been successful in identifying over 80 common and rare loci significantly associated with AD [10–17]. *APOE* and these other variants do not explain all genetic liability for AD. The hope is that with larger GWAS sample sizes, not only more risk loci will be identified, but also a larger proportion of total heritability will be explained. The amount of heritability still remaining to be found is under debate.

Heritability analyses were largely designed for the analysis of disorders of children and early adulthood in which both case and control designations have some certainty due to early in life onset and therefore were not influenced by age. Unfortunately, in AD these characteristics do not apply. The clinical diagnosis of AD is not particularly accurate, and the age dependence of the disease causes both obvious and subtle problems with analysis. For example, Beach et al. [4] show that 23.4% of people did not have frequent neuritic plaque density, despite their positive clinical diagnoses. Escott-Price et al. [5] estimate misdiagnosis rate up to 36% in controls unscreened for the APOE genotype, and up to 29% when E3 homozygous subjects are used as controls in clinical studies. The most important problem in estimating heritability is that an individual’s genetic loading for disease remains the same at any age, but the prevalence of AD is dependent on age. AD risk increases with age, people at the highest risk develop disease earlier, and therefore risk allele frequency decreases with age [18, 19]. Thus, heritability estimates are age dependent [20] and for reliable assessment at any individual age, it is necessary for cases and controls to be age matched. It is also possible that there will be some differences in the heritability of disease between populations, related to different haplotype length and to the presence/absence of rare mutations in the population e.g. the presenilin mutation (E280A) in Antioquia, Colombia [20]. All the above is reflected in widely different SNP-based heritability estimates across different datasets in AD, from as high as 53% [21] to as low as 3% [16]. The latter is unlikely to be true as the *APOE* gene alone explains 4% of the variance when studying incident AD [22]. Estimates are likely to be low due to LDSC underestimating heritability from summary [23]. Table 1 shows the heritability estimates for a number of different large scale studies [24].

**Table 1:** 

Publication	Sample Size	Narrow sense heritability on 5% liability scale
Harold et al. 2009	11,025	17%
Corneveaux et al. 2010	1,594	42%
Naj et al. 2011	21,165	25%
Lambert et al. 2013	54,162	9%
Marioni et al. 2018	368,440	3%
Kunkle et al. 2019	63,926	7%
Jansen et al. 2019	455,258	6% (without UK Biobank), 2% (with UK Biobank)
Rojas et al. 2021	409,435	3%
Wightman et al. 2021	1,126,563	3%
Bellenguez et al. 2022	487,551	3%

The variability of the reported heritability estimates arise from various sources, related to the populations studied and technical issues. The differences in heritability estimates may either be on the observed scale i.e. for the proportion of cases and controls as in the sample, or on the liability scale, i.e. assuming a disease prevalence in a particular population, which varies depending on the age group and population where the prevalence has been reported. For example, 2% lifetime prevalence was reported in the US in 2019 [25], 3% in 2020 in individuals aged 65–74 in the US [26], 5% lifetime prevalence in Europeans from a meta-analysis of multiple studies [27], 17% in 2020 in individuals aged 75–84 in the US [26], 32% in 2020 in individuals aged 85+ in the US [26]. Prevalence is not well-suited to late-onset disorders since this varies with age, with the risk of AD increasing substantially when reaching oldest ages [28]. For heritability analyses, the issue of prevalence is mostly resolved when using an age-matched cohort with relatively narrow age ranges in cases and controls. Age-related prevalence needs to be accounted for when computing heritability estimates.

The main aim of this study is to determine late onset AD heritability in a variety of AD data cohorts to understand the variability introduced by the liability model and age and evaluate whether consistent estimates can be determined for AD SNP-based heritability. Current methods do not allow age-related prevalence to be accounted for in one analysis, therefore, we computed heritability on the liability scale using three thresholds; 2, 5 and 15%. The convergence of the results across the thresholds will demonstrate that existing methods are appropriate when prevalence is correctly specified for the age of study participants. Next, we sought to utilise heritability estimates to give insights regarding where in the genome we should search for missing heritability, by investigating a gene-set specific to microglia which are known to be important in AD pathology. For this purpose, we investigate the proportion of heritability which can be explained using SNPs overlapping a specific gene-set related to microglia. We assess the proportion of heritability explained by this gene-set in comparison to the total heritability in the sample and compare this to the proportion of SNPs which explain this heritability.

## 2. Results

### 2.1 Cohort heritability estimates

**Fig 1:**
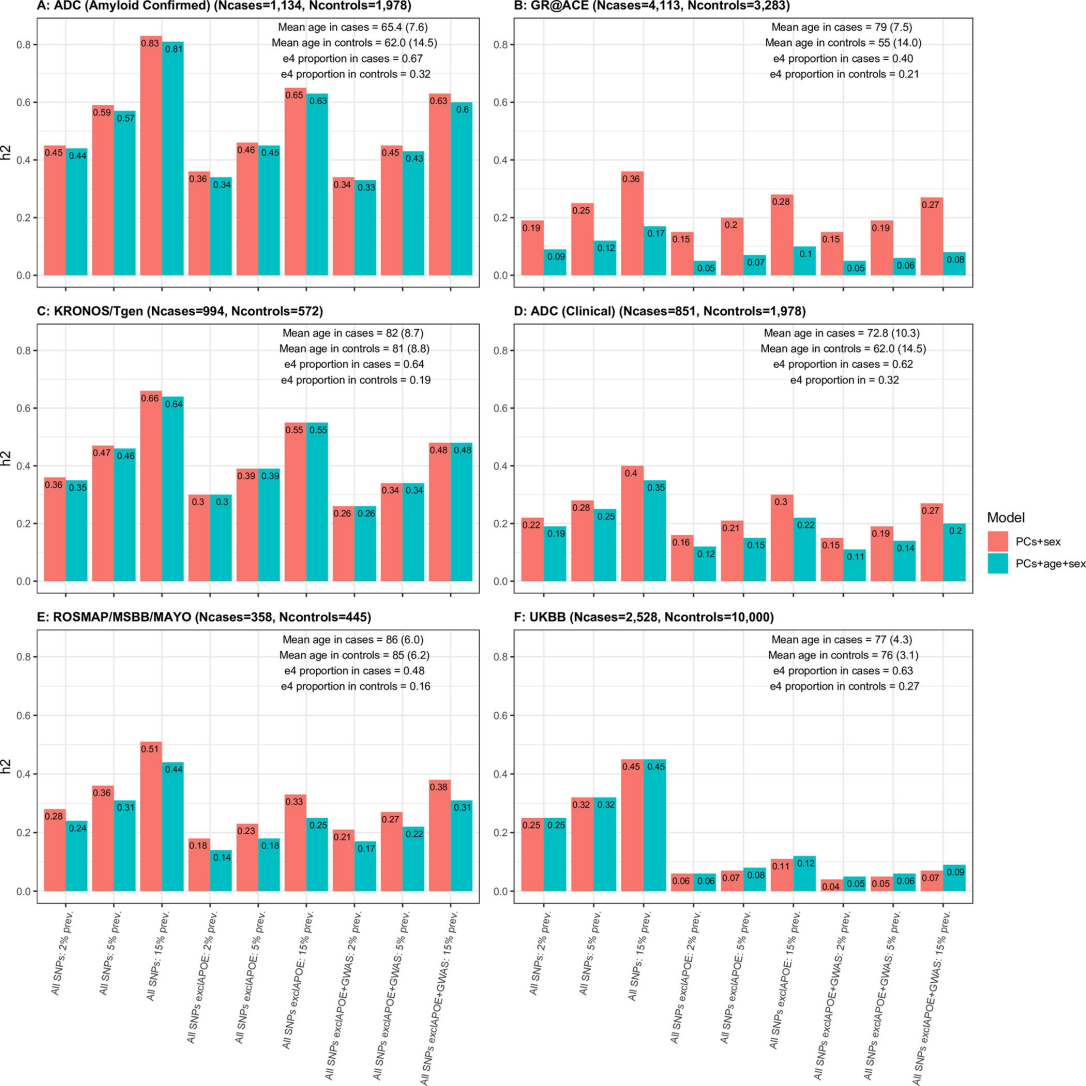
Heritability Estimates for AD prevalence of 2%, 5%, 15% in A) ADC with amyloid confirmed AD cases, B) GR@ACE, C) KRONOS/Tgen, D) ADC with clinical AD cases, E) ROSMAP/MSBB/MAYO, F) UKBB AD cases with controls aged 70+. Two models are considered: estimates adjusted for PCs and sex and PCs, sex and age.

First we present results for the heritability estimates calculated on the liability threshold with AD prevalence of 2%, 5% and 15% in all datasets; for A) ADC with amyloid confirmed AD cases, B) GR@ACE, C) KRONOS/Tgen, D) ADC with clinical AD cases, E) ROSMAP/MSBB/MAYO and F) UKBB with controls aged 70+, see Fig 1.

The results presented in Fig 1 show great variability in the heritability estimates even within the same liability threshold analyses (all estimates from all analyses can be observed in S1-S6 Tables in S1 File). When age is added as a covariate to an age mis-matched study (see e.g. (Fig 1B)), the estimates of heritability drop substantially, whereas in age-matched, pathologically confirmed cohorts of cases and controls, the heritability remains almost unchanged (see e.g. (Fig 1A and 1C)). Since age is a proxy of AD, adjusting for age in age mis-matched cohorts is biasing analyses towards the null hypothesis. Age mis-matched cohorts also cause issues in results because the prevalence of age-related disorders change with age, and therefore, the adjustment to the liability scale will not be appropriate for ages of cases or controls.

The heritability estimates decrease by 12% on average when the *APOE* region is removed and decrease ~1% further when the 0.5MB regions around GWAS index SNPs are additionally excluded. The largest decrease of more than 25% is observed in the UK Biobank cohort (Fig 1F) after removal of the *APOE* region.

In GR@ACE, the analysis was restricted to AD cases diagnosed with probable AD at both first and second diagnoses (N = 1,851). The heritability estimates increased for all prevalences by 10% [SD = 3%] on average to 0.27, 0.35 and 0.49 for 2, 5 and 15% prevalences respectively, when adjusted for PCs and sex. Thus making estimates in this sample more comparable to the other cohorts.

We investigate the impact of the age of controls in UKBB by using four age bins for the control subset (≤60, 60–70, 70–80 and 80+ years old). It is seen from S1 Fig in S1 File that heritability estimates are fairly consistent for controls at all ages, with estimates being slightly increased for the group with the youngest controls (≤60 years old). The model adjusted for PCs, sex and age did not converge in the two youngest control groups since there was little overlap in age distributions between cases and controls.

The p-value of the heritability estimates were directly linked to the size of the cohorts (see S2 Fig and S1, S4-S6 Tables in S1 File). In the KRONOS/Tgen dataset (N = 1,566) the significance reaches p = 3.22×10^−3^ when all SNPs were included and p = 0.02 after exclusion of *APOE* and GWAS regions. In ADC (clinical) and ROSMAP/MSBB/MAYO all heritability estimates are non-significant for all models, see S2, S3 Tables in S1 File.

The heritability estimates in cohorts with pathologically/amyloid confirmed diagnosis (Fig 1, left plots) are higher (0.36–0.59) compared to cohorts with a clinical diagnosis only (Fig 1, right plots) (h^2^ = 0.25–0.34). This is expected as a pathologically/amyloid confirmed diagnosis is more accurate than a clinical diagnosis of AD which may contain up to 30% of misdiagnosed individuals [4, 5]. Heritability estimates adjusting for PCs only are very similar to those adjusting for PCs and sex, see S3 Fig in S1 File.

As noted above, the additional adjustment for age has little impact on heritability estimates in the pathologically/amyloid confirmed data but reduces the estimates in the GR@ACE data by more than 13%. This result suggests that the decrease in heritability estimate could be mainly attributed to the difference in age distribution between cases and controls. Although it is tempting to adjust for age by including it as a covariate, it is difficult to do this effectively. If there is a systematic age difference between cases and controls, the age covariate largely absorbs the disease status effect, and the analysis is biased towards the null hypothesis. This suggests that the observed heritability should be estimated without adjustment for age but accounted for when transforming to the liability scale. For example, in GR@ACE data, the mean cases’ age (79 [SD = 7.5]) is above the average onset of e44 and e4 carriers (which is 68 and 76, respectively [29], whereas the controls are below this age 54.5 [SD = 14.0]. Therefore, if they live until their 80s, more than 15% of controls could develop AD, indicating that they have genetic liability to the disease.

### 2.2 Gene-set heritability estimates

Table 2 demonstrates the proportion of heritability and number of SNPs in the microglia gene-set compared to those including all SNPs for ADC with amyloid confirmed AD cases, GR@ACE, KRONOS/Tgen, ADC with clinical AD cases, ROSMAP/MSBB/MAYO and UKBB with controls aged 70+. The absolute heritability estimates adjusted for PCs and sex for each cohort can be seen in S4 Fig in S1 File.

**Table 2:** Proportion of heritability and SNPs explained by a microglia gene-set in all data cohorts across all disease prevalences.

Data Cohort	Sample Size	Microglia	
		Proportion of h2	Proportion of SNPs
KRONOS/Tgen	1,566	0.64	0.028
ROSMAP/MSBB/MAYO	803	0.68–0.69	0.030
GR@ACE	7,396	0.50–0.53	0.032
UKBB (70+ controls)	12,528	0.80–0.82	0.032
ADC (amyloid confirmed)	3,112	0.67–0.69	0.032
ADC (clinical)	2,829	0.91–0.93	0.032

Heritability estimates adjusted for PCs and sex.

It can be seen that by selecting cell-type specific SNPs, a substantial proportion of heritability is explained using fewer SNPs (approximately 3% of SNPs in the microglia gene-set). The proportion of heritability explained for the microglia gene-set was 68–69% in ROSMAP/MSBB/MAYO, 80–82% in UKBB, 64% in KRONOS/Tgen, 67–69% for amyloid confirmed ADC and 91–93% for clinical cases ADC. The range of values represent the proportions across all AD disease prevalences.

In general, the microglia gene-set has lower heritability estimates compared to all SNPs, however, the reduction is not proportional to the reduction in the number of SNPs, see Table 2. It can be seen in S4 Fig in S1 File that the microglia gene-set produces comparable heritability estimates with the model excluding the *APOE* region. We also present heritability estimates for the microglia gene-set with the same parameters as in S4 Fig in S1 File but adjusted for PCs, sex and age in S5 Fig and S1-S6 Tables in S1 File. Thus, despite this gene-set utilising a much-reduced number of SNPs, it is able to explain a substantial proportion of AD heritability.

We also investigate the impact of the age of controls in UKBB by using four age bins for the control subset (≤60, 60–70, 70–80 and 80+ years old) on the proportion of heritability explained by microglia SNPs. The results are seen in S6 Fig in S1 File, the microglia gene-set explains a consistent proportion of heritability across all control age groups (75–87%).

## 3. Discussion

To date, reported SNP-based heritability estimates in AD have been very varied across different datasets and methodologies. We studied five different cohorts and harmonized analytical methods to estimate SNP-based heritability. We estimate that the SNP-based heritability is between 36% and 59% in pathologically or CSF confirmed AD and 25% to 32% in clinically assessed cohorts when assuming, for the purposes of the heritability model, AD prevalence of 5%. The regions related to microglial genes (only 3% of SNPs) explain between 50% and 93% of the SNP based heritability. This shows the importance of further development of biologically relevant AD gene-sets/pathways that could reduce the signal to noise ratio by highlighting the most influential SNPs/genes in AD. Novel loci are most likely to be expected in these regions.

These heritability estimates have implications for understanding AD risk. For example, Zhang et al. [30] proposed that AD is oligogenic (based on <100 SNPs) based on simulations assuming 9% narrow-sense heritability excluding *APOE*. The larger heritability estimates reported here will change conclusions about the number of loci contributing to AD risk.

We studied the effects of age and *APOE* on heritability estimates. The results show that heritability estimates are systematically reduced when the *APOE* region is excluded. The reduction varies across cohorts with the largest decrease in UK Biobank, likely due to the age of the UK Biobank cases which is ~76–77 which is the age at onset for e4 carriers [29]. When GWAS hits are additionally excluded, the heritability estimates reduce further but only by a small amount.

The inclusion of age as a covariate clearly has a large impact on the heritability estimates for data cohorts where the mean age of cases and controls differs substantially. Where there is little difference in age between cases and controls, heritability estimates do not change. Based on these observations we recommend that age should not be used as covariate, since a difference in age distribution between cases and controls will lead to adjustment for ‘caseness’ by biasing the analysis towards the null, and therefore reducing the heritability estimates significantly. Instead, we suggest that the genetic architecture of AD is different depending on age at clinical onset. Indeed, it is known that very early AD cases (aged 30–50) are mostly attributed to rare highly penetrant mutations in *APP* and *PSEN* genes. The disease prevalence at this age in the population is then close to the frequencies of these risk alleles (<1%). *APOE* e44 carriers have age at onset of about 68, and the disease prevalence at this age is likely to be around or slightly larger than e44 frequency (~2–3%), due to the variation in the age at onset of e4 heterozygotes and non-carriers. The mean age of clinical onset of e4 non-carriers is ~84 years of age [29]. The disease prevalence at this age is reported as something between 17–32% [26]. The disease at this age is likely to be attributed to a large number of common SNPs associated with a variety of disease development mechanisms, including comorbid disorders. It is worth noting that the density of AD pathology required for an AD diagnosis is less as age increases [31]. Furthermore, several studies have shown age dependent association of AD polygenic risk score (PRS) with Alzheimer’s disease and cognitive function, with almost no association in those with age below 50 years [32], with GWAS significant SNP-based PRS association in samples with mean age 60–65 [17, 33], and with genome-wide PRS association in samples aged 65+ [32, 34–36]. Mars et al. [19] showed 4–9 years earlier age at onset with high polygenic risk in other age-related diseases (type 2 diabetes, atrial fibrillation, breast cancer and prostate cancer), but they do not explain the increasing prevalence of these diseases with age in the population, as earlier onset implies earlier mortality. A simulation study [18] showed that the AD risk increases with age, and therefore, that risk allele frequency decreased with age due to people at the highest risk developing disease earlier [18]. In this circumstance it is perhaps not surprising that the architecture of genetic risk is different at different ages. Therefore, we suggest that for neurodegenerative disorders, the heritability estimates on the liability scale should be adjusted for the *age-related* prevalence of cases. If the controls are not screened for the disease, the proportion of cases in the sample needs to be uplifted to account for the genetic liability for the disease of individuals who do not yet show symptoms, and the observed heritability adjusted accordingly before transforming it to the liability scale. For example, the observed heritability in the GR@ACE data was estimated $h_{o}^{2}$ = 0.30 (see S1 Table in S1 File) with the proportion of cases *P* = 0.56 with mean age 84 years. Assuming that 15% of controls (who are on average 54 years old) will develop the disease given time, the actual proportion of cases is *P*_actual_ = 0.62, and therefore $h_{l}^{2}$ = 0.38, (see equation (23) in [37]), which is 2% higher than shown in Fig 1B (“All SNPs: 15% prev”). In contrast, in the ADC—amyloid confirmed sample (mean age in cases 65.4), the observed heritability does not need to be adjusted (as ages of cases and controls are similar), and the SNP-based heritability on the liability scale should be reported as $h_{l}^{2}$ = 0.45 (Fig 1B (“All SNPs: 2% prev”)).

The limitations of this study are that some of the datasets have small sample sizes, therefore have broad confidence intervals which impacts the statistical significance of the heritability estimates. Another limitation is that all cohorts investigated are Caucasian, but the GR@ACE cohort has a different genetic architecture compared to the other cohorts (Spain compared to North Caucasian) [38]. AD diagnostic criteria in the cohorts of this study are different, varying from pathologically confirmed, amyloid confirmed to clinical diagnosis which introduces noise in the heritability estimates.

In conclusion, for late onset diseases such as AD, the heritability cannot be represented as a single number, but in fact depends upon the age of the cases and controls in the sample where the heritability is to be determined. We also determined that a major fraction of AD heritability independent of cohort age is attributed to the microglia gene-set, highlighting the importance of biologically relevant gene-sets in AD development.

## 4. Methods

The cohorts which were investigated are 1) Genome Research at Fundacio ACE (GR@ACE) [39], 2) KRONOS/Tgen [40–43], 3) Religious Orders Study and the Rush Memory and Aging Project (ROSMAP) data [44–46], The Mount Sinai Brain Bank (MSBB), MAYO Clinic Brain Bank (MAYO), 4) UK Biobank (UKBB) data [47] and 5) the Amsterdam Dementia Cohort (ADC) [48]. These data vary in terms of sample size, age, the definition of late onset AD and control phenotypes (e.g. pathologically confirmed or clinically defined AD cases; age-matched or population cohort controls). All samples are anonymised and therefore it is not possible to identify any individuals.

Heritability was computed in each series independently a) for all available SNPs in each data cohort, b) for all SNPs excluding the *APOE* region (chr19: 44.4–46.5Mb), and c) for all SNPs but with both *APOE* SNPs and SNPs within 0.5Mb of previously reported genome-wide association study (GWAS) hits excluded. For comparability with other studies (e.g. [30]), the estimates were adjusted to the liability scale based on AD disease prevalence in the population (5% [27]). We however present and discuss the results for 2%, 5% and 15% prevalence.

### 4.1 Population description

The GR@ACE data [39] consists of 4,113 cases and 3,283 controls. AD cases are classified as individuals with dementia who were diagnosed with either possible or probable AD at any time. Written informed consent was obtained from all participants. The Ethics and Scientific Committees have approved this research protocol (Acta 25/2016. Ethics Committee. H. Clinic i Provincial, Barcelona, Spain).

The KRONOS/Tgen dataset is obtained from 21 National Alzheimer’s Coordinating Center (NACC) brain banks and from the Miami Brain Bank as previously described [40–43]. The cohort consists of 994 AD cases and 572 controls of European descent. This work is declared not human-subjects research and is IRB exempt under regulation 45 CFR 46.

ROSMAP [44–46], MSBB (The Mount Sinai Brain Bank) and The Mayo Clinic Brain Bank (MAYO) have been whole-genome sequenced, harmonised and analysed together. This sample contains 803 individuals; 358 AD cases and 445 controls. The parent studies and sub-studies were all approved by an Institutional Review Board of Rush University Medical Center and all participants signed an informed consent, Anatomical Gift Act, and a repository consent to share data and biospecimens.

The UKBB is a large prospective cohort of individuals from the UK recruited between 2006–2010 [47]. Inclusion criteria was for cases -all individuals who were diagnosed with AD based on ICD-10 code F00 or G30, N = 2,528 and for controls -a subset of 10,000 individuals with no AD or dementia diagnosis who were aged over 70 (UKBB (controls 70+)). All participants provided informed written consent to participate in UK Biobank. Ethical approval was granted to the UK Biobank project by the North West Multi-Centre Ethics committee. Data were released to us after application project reference 13310.

A secondary analysis to investigate the impact of the age of controls was carried out using four different control subsets; 1) aged ≤60 years old, 2) aged 60–70 years old, 3) aged 70–80 years old and 4) aged 80+ years old.

The Amsterdam Dementia cohort (ADC) data [48, 49] is a cohort of AD cases and controls, consisting of 1,985 cases (1,134 CSF confirmed and 851 clinically diagnosed) and 1,978 controls. All patients gave informed consent. The local Medical Ethics Committee has approved a general protocol for biobanking and using the clinical data for research purposes.

Detailed information and demographics for all the cohorts can be found in Table 3 and S1 File.

**Table 3:** Summary of demographics for all cohorts.

Data	Demographics	Cases	Controls
GR@ACE	N	4113	3283
	Age at onset/interview [SD]	79 [7.5]	54.5 [14.0]
	Sex [M/F/NA]	1256/2856/1	1676/1605/2
KRONOS/Tgen	N	994	572
	Age of death [SD]	81.9 [8.7]	81 [8.8]
	Sex [M/F]	361/633	355/217
ROSMAP/MSBB/MAYO	N	358	445
	Age of death [SD]	85.9 [6.0]	84.5 [6.2]
	Sex [M/F]	100/258	167/278
UKBB	N	2528	10,000
	Age at interview [SD]	76.8 [4.3]	75.2 [3.3]
	Sex [M/F]	1227/1301	4706/5294
ADC	N	1985 (1134 CSF, 851 clinical)	1978
	Age at interview [SD]	65.4 [7.6] (CSF) 72.8 [10.3] (clinical)	62.0 [14.5]
	Sex [M/F]	540/594 (CSF) 373/478 (clinical)	1031/947

### 4.2 Heritability estimates

Heritability estimates are computed using the Genome-wide Complex Trait Analysis (GCTA) [50, 51] software to estimate the proportion of phenotypic variance explained by SNPs. GCTA software was chosen as the primary approach for calculation of heritability estimates since a) individual genotypes were available to us, and b) when a large proportion of the SNP-based heritability is explained by a single variant, the genome-based restricted maximum likelihood, implemented in GCTA, is unbiased whereas the alternative approach (LDScore regression [52]) in this case provides systematically lower estimates [53]).

The restricted maximum likelihood (GREML-LDMS) analysis was used to estimate SNP-based heritability whilst correcting for LD bias, by splitting data into LD quartiles and stratifying SNPs based on the segment-based LD score and MAF = 0.05. For this analysis, a region of 200kb was used to compute the segment-based LD score. The heritability was estimated in two scenarios 1) adjusting for principal components (PCs) and sex, and 2) for PCs, age and sex. The GR@ACE and KRONOS/Tgen data were adjusted for 5 PCs; the ROSMAP/MSBB/MAYO dataset is adjusted for 8 PCs, UKBB is adjusted for 15 PCs and the ADC is adjusted for 10 PCs, determined from PC plots.

The GCTA software was applied to the five datasets separately, using a) all available SNPs, b) excluding the *APOE* region (chr19:44.4–46.5Mb), and c) excluding SNPs in the *APOE* region and those within 0.5Mb of known GWAS hits [54]. Observed heritability estimates were re-scaled to the liability threshold based on 2%, 5% and 15% prevalences which represent a range of prevalences previously published [25–27].

### 4.3 Gene-sets

A number of biological gene-sets have been defined which may enable the AD genetic signal to be focused to specific biological functions. We investigated the proportion of heritability explained by SNPs in genes related to microglia. [55] defined microglia regions based on GWAS signatures and epigenetic/gene regulatory data. [56] have redefined the list of SNPs to include established regulatory regions of the genes. We have used SNPs within these regions and heritability based on these SNPs was computed to compare heritability in each data cohort.

1. Research Center and Memory clinic. ACE Alzheimer Center Barcelona, Universitat Internacional de Catalunya, Spain.
2. CIBERNED, Center for Networked Biomedical Research on Neurodegenerative Diseases, National Institute of Health Carlos III, Ministry of Economy and Competitiveness, Spain,
3. Dep. of Surgery, Biochemistry and Molecular Biology, School of Medicine. University of Málaga. Málaga, Spain,
4. Grupo de Medicina Xenómica, Centro Nacional de Genotipado (CEGEN-PRB3-ISCIII). Universidad de Santiago de Compostela, Santiago de Compostela, Spain.
5. Fundación Pública Galega de Medicina Xenómica- CIBERER-IDIS, Santiago de Compostela, Spain.
6. Centro de Investigación Biomédica en Red de Diabetes y Enfermedades Metabólicas Asociadas, CIBERDEM, Spain, Hospital Clínico San Carlos, Madrid, Spain,
7. CAEBI. Centro Andaluz de Estudios Bioinformáticos, Sevilla, Spain
8. Unidad Clínica de Enfermedades Infecciosas y Microbiología. Hospital Universitario de Valme, Sevilla, Spain.

## Supporting information

S1 File (DOCX)

S1 Data (CSV)
