# Supplementary material for: What does heritability of Alzheimer’s disease represent?
Source: PLoS One. 2023 Apr 28;18(4):e0281440. doi: 10.1371/journal.pone.0281440 (PMC10146480; doi:10.1371/journal.pone.0281440)
Supplement: S1 File — (DOCX) [file pone.0281440.s001.docx]

# Supplementary material

### Population description

#### Genome Research at Fundacio ACE (GR@ACE)

The GR@ACE data (Moreno-Grau et al., 2019) is a genome-wide association study of individuals recruited in Spain, consisting of 4,113 cases and 3,283 controls. AD cases are classified as individuals with dementia who were diagnosed with either possible or probable AD at any time. There were 1,851 AD cases classified as having probable AD at both first and second diagnoses. The controls in this sample are taken from a population cohort. The mean age at onset in cases is 79 [SD=7.5] years and the mean age at interview in controls is 55 [SD=14.0] years. This data was imputed using the Michigan Imputation Server (Das et al., 2016) using the Haplotype Reference Consortium panel, and SNPs with an INFO score less than 0.7 were removed.

#### KRONOS/Tgen

The KRONOS/Tgen dataset is obtained from 21 National Alzheimer’s Coordinating Center (NACC) brain banks and from the Miami Brain Bank as previously described (Corneveaux et al., 2010; Myers et al., 2007; Petyuk et al., 2018; Webster et al., 2009). The criteria for inclusion were as follows: self-defined ethnicity of European descent (in an attempt to control for the known allele frequency differences between ethnic groups), neuropathologically confirmed AD or no neuropathology present, and age of death greater than 65. Neuropathological diagnosis was defined by board-certified neuropathologists according to the standard NACC protocols (Beekly et al., 2004). Samples derived from subjects with a clinical history of stroke, cerebrovascular disease, Lewy bodies, or comorbidity with any other known neurological disease were excluded. Alzheimer’s disease or control neuropathology was confirmed by plaque and tangle assessment with 45% of the entire series undergoing Braak staging (Braak & Braak, 1995). The cohort consists of 994 AD

cases and 572 controls of European descent. The mean age in AD cases and controls is similar in this sample, 82 [SD=8.7] and 81 [SD=8.8] years old respectively. Samples were de-identified before receipt, and the study met human studies institutional review board and HIPPA regulations. This work is declared not human-subjects research and is IRB exempt under regulation 45 CFR 46. This data was imputed using the Michigan Imputation Server (Das et al., 2016) using the Haplotype Reference Consortium panel, and SNPs with an INFO score less than 0.8 were removed.

#### ROSMAP/MSBB/MAYO

The next three datasets were downloaded from the AMP-AD portal via the Synapse platform and [https://www.radc.rush.edu](about:blank). ROSMAP (Bennett et al., 2011; Bennett et al., 2018; Bennett et al., 2012) is an ongoing longitudinal cohort study of ageing and AD (syn3191087, syn10901595). The MSBB (The Mount Sinai Brain Bank) study has generated gene expression, genomic variant, proteomic and neuropathological data from brain specimens (syn6101474, syn10901600). The Mayo Clinic Brain Bank (MAYO) is a post-mortem cohort that contains neuropathological, genetic, biochemistry and cell biology data (syn3817650, syn10901601). Quality-control analysis have been performed on the combined dataset, see details in (Leonenko et al., 2021). AD cases are defined by their clinical definition for AD and Braak score of 5 or 6, controls are defined as those without a clinical definition and Braak score less than or equal to 4. This sample contains 803 individuals; 358 AD cases and 445 controls, the mean age of death in cases is 86 [SD=6.0] years and 85 [SD=6.2] years in controls. These datasets have been whole-genome sequenced, harmonised and analysed together.

#### UK Biobank (UKBB)

The UKBB is a large prospective cohort of approximately 500,000 individuals from the UK containing extensive phenotypic and genotypic data which are still being collected (Sudlow et al., 2015). A wealth of lifestyle, sociodemographic, medical, and family history information were collected through a computer-based, self-completed questionnaire during the first assessment visit, along with physical measurements, biological samples, and cognitive testing. Additional information can be found at https://www.ukbiobank.ac.uk/.

This study used the UKBB data under UKBB approval for application #13310 “Impact of genetic and environmental associations on neuropsychiatric disorders and associated phenotypes”. For the present study, participants were identified as being diagnosed with AD based on diagnosis codes across all hospital inpatient records. Diagnoses are coded according to the International Classification of Diseases version-10 (ICD-10). For this study we took a subset of individuals from UKBB to ensure that cases and controls are matched by age. Inclusion criteria was for cases -all individuals who were diagnosed with AD based on ICD-10 code F00 or G30, N=2,528 and for controls -a subset of 10,000 individuals with no AD or dementia diagnosis who were aged over 70 (UKBB (controls 70+)). The mean age at interview in cases is 77 [SD=4.3] and in 70+ controls is 76 [SD=3.1] years old.

A secondary analysis to investigate the impact of the age of controls was carried out using four different control subsets; 1) aged ≤60 years old, 2) aged 60-70 years old, 3) aged 70-80 years old and 4) aged 80+ years old. In these subsets the mean age is 1) 56.4 [SD=2.5], 2) 65.8 [SD=2.9], 3) 75.1 [SD=2.8] and 4) 81.4 [SD=0.6] years, respectively.

#### Amsterdam Dementia Cohort (ADC)

The Amsterdam Dementia cohort (ADC) data (Tesi et al., 2020; van der Flier & Scheltens, 2018) is a cohort of AD cases and controls, consisting of 1,985 cases (1,134 CSF confirmed and 851 clinically diagnosed) and 1,978 controls. The mean age in AD CSF confirmed cases is 65.4 [SD=7.6] and in clinically diagnosed cases is 72.8 [SD=10.3]. The mean age of controls is 62.0 [SD=14.5] years. The same group of controls was used for heritability estimates for the two independent case samples.

#### Data Quality Control

All data underwent standard quality control checks in each dataset separately using PLINK (Chang et al., 2015). Related individuals were removed based on identity by descent with PI_HAT > 0.2 and samples with heterozygosity (HET > ±0.1) were removed. SNPs with a missing data proportion > 5%, those not in Hardy-Weinberg equilibrium (p≤10^-6^). We restricted our main analysis to SNPs with minor allele frequency (MAF) threshold of 0.05 after imputation. Only individuals of European ancestry were included in this analysis, based on a comparison of samples against the 1000 Genomes data.

These cohorts were considered independently and not meta-analysed together since each cohort varies in terms of phenotype definition, genetic architecture, SNP availability and age of cases and controls.

Supplementary Figure 1- Heritability Estimates in UKBB with controls of different ages adjusted for PCs (red), PCs+sex (green) and PCs+sex+age (blue).

Supplementary Figure 2- Relationship between sample size and p-values from heritability estimates. Based on heritability analyses adjusted for PCs+sex, and including all SNPs.

Supplementary Figure 3- Heritability Estimates adjusted for PCs only (red), PCs+sex (green) and PCs+sex+age (blue) for A) ADC with amyloid confirmed AD cases, B) GR@ACE, C) KRONOS/Tgen, D) ADC with clinical AD cases, E) ROSMAP/MSBB/MAYO, F) UKBB with controls aged 70+.

Supplementary Figure 4- Heritability Estimates adjusted for PCs+sex in gene-sets for A) ADC with amyloid confirmed AD cases, B) GR@ACE, C) KRONOS/Tgen, D) ADC with clinical AD cases, E) ROSMAP/MSBB/MAYO, F) UKBB with controls aged 70+.

Supplementary Figure 5- Heritability Estimates adjusted for PCs+sex+age in gene-sets for A) ADC with amyloid confirmed AD cases, B) GR@ACE, C) KRONOS/Tgen, D) ADC with clinical AD cases, E) ROSMAP/MSBB/MAYO, F) UKBB with controls aged 70+.

Supplementary Figure 6- Heritability Estimates in UKBB with controls of different ages for All SNPs and Microglia geneset SNPs adjusted for PCs+sex.

Supplementary Table 1- Heritability Estimates in GR@ACE

| **Model** | **Liability** | **PCs+sex** | | **PCs+sex+age** | |
| --- | --- | --- | --- | --- | --- |
|  |  | **H2** | **p-val** | **H2** | **p-val** |
| All SNPs | Observed | 0.30 | 7.07E-06 | 0.14 | 4.32E-03 |
| All SNPs | 2% | 0.19 | 7.07E-06 | 0.09 | 4.32E-03 |
| All SNPs | 5% | 0.25 | 7.07E-06 | 0.12 | 4.32E-03 |
| All SNPs | 15% | 0.36 | 7.07E-06 | 0.17 | 4.32E-03 |
| All SNPs exclAPOE | Observed | 0.23 | 1.77E-03 | 0.08 | 1.07E-01 |
| All SNPs exclAPOE | 2% | 0.15 | 1.77E-03 | 0.05 | 1.07E-01 |
| All SNPs exclAPOE | 5% | 0.20 | 1.77E-03 | 0.07 | 1.07E-01 |
| All SNPs exclAPOE | 15% | 0.28 | 1.77E-03 | 0.10 | 1.07E-01 |
| All SNPs exclAPOE+GWAS | Observed | 0.23 | 2.05E-03 | 0.07 | 1.45E-01 |
| All SNPs exclAPOE+GWAS | 2% | 0.15 | 2.05E-03 | 0.05 | 1.45E-01 |
| All SNPs exclAPOE+GWAS | 5% | 0.19 | 2.05E-03 | 0.06 | 1.45E-01 |
| All SNPs exclAPOE+GWAS | 15% | 0.27 | 2.05E-03 | 0.07 | 1.45E-01 |
| Microglia | Observed | 0.15 | 6.62E-08 | 0.07 | 3.34E-05 |
| Microglia | 2% | 0.10 | 6.62E-08 | 0.08 | 3.34E-05 |
| Microglia | 5% | 0.13 | 6.62E-08 | 0.06 | 3.34E-05 |
| Microglia | 15% | 0.19 | 6.62E-08 | 0.09 | 3.34E-05 |

Supplementary Table 2- Heritability Estimates in ROSMAP/MSBB/MAYO

| **Model** | **Liability** | **PCs+sex** | | **PCs+sex+age** | |
| --- | --- | --- | --- | --- | --- |
|  |  | **H2** | **p-val** | **H2** | **p-val** |
| All SNPs | Observed | 0.42 | 3.58E-01 | 0.37 | 3.05E-01 |
| All SNPs | 2% | 0.28 | 3.58E-01 | 0.24 | 3.05E-01 |
| All SNPs | 5% | 0.36 | 3.58E-01 | 0.31 | 3.05E-01 |
| All SNPs | 15% | 0.51 | 3.58E-01 | 0.44 | 3.05E-01 |
| All SNPs exclAPOE | Observed | 0.27 | 3.01E-01 | 0.21 | 4.18E-01 |
| All SNPs exclAPOE | 2% | 0.18 | 3.01E-01 | 0.14 | 4.18E-01 |
| All SNPs exclAPOE | 5% | 0.24 | 3.01E-01 | 0.18 | 4.18E-01 |
| All SNPs exclAPOE | 15% | 0.33 | 3.01E-01 | 0.25 | 4.18E-01 |
| All SNPs exclAPOE+GWAS | Observed | 0.31 | 3.82E-01 | 0.26 | 4.11E-01 |
| All SNPs exclAPOE+GWAS | 2% | 0.21 | 3.82E-01 | 0.17 | 4.11E-01 |
| All SNPs exclAPOE+GWAS | 5% | 0.27 | 3.82E-01 | 0.22 | 4.11E-01 |
| All SNPs exclAPOE+GWAS | 15% | 0.38 | 3.82E-01 | 0.31 | 4.11E-01 |
| Microglia | Observed | 0.29 | 5.07E-02 | 0.30 | 4.47E-02 |
| Microglia | 2% | 0.19 | 5.07E-02 | 0.19 | 4.47E-02 |
| Microglia | 5% | 0.25 | 5.07E-02 | 0.26 | 4.47E-02 |
| Microglia | 15% | 0.35 | 5.07E-02 | 0.36 | 4.47E-02 |

Supplementary Table 3- Heritability Estimates in KRONOS/Tgen

| **Model** | **Liability** | **PCs+sex** | | **PCs+sex+age** | |
| --- | --- | --- | --- | --- | --- |
|  |  | **H2** | **p-val** | **H2** | **p-val** |
| All SNPs | Observed | 0.51 | 3.22E-03 | 0.50 | 2.14E-03 |
| All SNPs | 2% | 0.36 | 3.22E-03 | 0.35 | 2.14E-03 |
| All SNPs | 5% | 0.47 | 3.22E-03 | 0.46 | 2.14E-03 |
| All SNPs | 15% | 0.66 | 3.22E-03 | 0.64 | 2.14E-03 |
| All SNPs exclAPOE | Observed | 0.43 | 1.14E-02 | 0.43 | 1.12E-02 |
| All SNPs exclAPOE | 2% | 0.30 | 1.14E-02 | 0.30 | 1.12E-02 |
| All SNPs exclAPOE | 5% | 0.39 | 1.14E-02 | 0.39 | 1.12E-02 |
| All SNPs exclAPOE | 15% | 0.55 | 1.14E-02 | 0.55 | 1.12E-02 |
| All SNPs exclAPOE+GWAS | Observed | 0.37 | 2.49E-02 | 0.37 | 2.39E-02 |
| All SNPs exclAPOE+GWAS | 2% | 0.26 | 2.49E-02 | 0.26 | 2.39E-02 |
| All SNPs exclAPOE+GWAS | 5% | 0.34 | 2.49E-02 | 0.34 | 2.39E-02 |
| All SNPs exclAPOE+GWAS | 15% | 0.48 | 2.49E-02 | 0.48 | 2.39E-02 |
| Microglia | Observed | 0.33 | 1.51E-02 | 0.34 | 1.29E-02 |
| Microglia | 2% | 0.23 | 1.51E-02 | 0.24 | 1.29E-02 |
| Microglia | 5% | 0.30 | 1.51E-02 | 0.31 | 1.29E-02 |
| Microglia | 15% | 0.42 | 1.51E-02 | 0.44 | 1.29E-02 |

Supplementary Table 4- Heritability Estimates in UKBB

| **Model** | **Liability** | **PCs+sex** | | **PCs+sex+age** | |
| --- | --- | --- | --- | --- | --- |
|  |  | **H2** | **p-val** | **H2** | **p-val** |
| All SNPs | Observed | 0.24 | 5.55E-17 | 0.25 | 3.89E-16 |
| All SNPs | 2% | 0.25 | 5.55E-17 | 0.25 | 3.89E-16 |
| All SNPs | 5% | 0.32 | 5.55E-17 | 0.32 | 3.89E-16 |
| All SNPs | 15% | 0.45 | 5.55E-17 | 0.45 | 3.89E-16 |
| All SNPs exclAPOE | Observed | 0.06 | 3.32E-01 | 0.06 | 3.64E-01 |
| All SNPs exclAPOE | 2% | 0.06 | 3.32E-01 | 0.06 | 3.64E-01 |
| All SNPs exclAPOE | 5% | 0.08 | 3.32E-01 | 0.08 | 3.64E-01 |
| All SNPs exclAPOE | 15% | 0.11 | 3.32E-01 | 0.12 | 3.64E-01 |
| All SNPs exclAPOE+GWAS | Observed | 0.04 | 4.31E-01 | 0.05 | 4.47E-01 |
| All SNPs exclAPOE+GWAS | 2% | 0.04 | 4.31E-01 | 0.05 | 4.47E-01 |
| All SNPs exclAPOE+GWAS | 5% | 0.05 | 4.31E-01 | 0.06 | 4.47E-01 |
| All SNPs exclAPOE+GWAS | 15% | 0.07 | 4.31E-01 | 0.09 | 4.47E-01 |
| Microglia | Observed | 0.20 | 1.50E-01 | 0.19 | 1.77E-01 |
| Microglia | 2% | 0.20 | 1.50E-01 | 0.20 | 1.77E-01 |
| Microglia | 5% | 0.26 | 1.50E-01 | 0.25 | 1.77E-01 |
| Microglia | 15% | 0.37 | 1.50E-01 | 0.36 | 1.77E-01 |

Supplementary Table 5- Heritability Estimates in ADC with Amyloid Confirmed AD Cases

| **Model** | **Liability** | **PCs+sex** | | **PCs+sex+age** | |
| --- | --- | --- | --- | --- | --- |
|  |  | **H2** | **p-val** | **H2** | **p-val** |
| All SNPs | Observed | 0.64 | 1.31E-08 | 0.63 | 2.36E-08 |
| All SNPs | 2% | 0.45 | 1.31E-08 | 0.44 | 2.36E-08 |
| All SNPs | 5% | 0.59 | 1.31E-08 | 0.57 | 2.36E-08 |
| All SNPs | 15% | 0.83 | 1.31E-08 | 0.81 | 2.36E-08 |
| All SNPs exclAPOE | Observed | 0.51 | 4.11E-05 | 0.49 | 7.35E-05 |
| All SNPs exclAPOE | 2% | 0.36 | 4.11E-05 | 0.35 | 7.35E-05 |
| All SNPs exclAPOE | 5% | 0.46 | 4.11E-05 | 0.45 | 7.35E-05 |
| All SNPs exclAPOE | 15% | 0.65 | 4.11E-05 | 0.63 | 7.35E-05 |
| All SNPs exclAPOE+GWAS | Observed | 0.49 | 5.43E-05 | 0.47 | 1.12E-04 |
| All SNPs exclAPOE+GWAS | 2% | 0.34 | 5.43E-05 | 0.33 | 1.12E-04 |
| All SNPs exclAPOE+GWAS | 5% | 0.45 | 5.43E-05 | 0.43 | 1.12E-04 |
| All SNPs exclAPOE+GWAS | 15% | 0.63 | 5.43E-05 | 0.61 | 1.12E-04 |
| Microglia | Observed | 0.43 | 3.79E-13 | 0.43 | 7.77E-13 |
| Microglia | 2% | 0.31 | 3.79E-13 | 0.31 | 7.77E-13 |
| Microglia | 5% | 0.40 | 3.79E-13 | 0.40 | 7.77E-13 |
| Microglia | 15% | 0.56 | 3.79E-13 | 0.56 | 7.77E-13 |

Supplementary Table 6- Heritability Estimates in ADC with Clinical AD Cases

| **Model** | **Liability** | **PCs+sex** | | **PCs+sex+age** | |
| --- | --- | --- | --- | --- | --- |
|  |  | **H2** | **p-val** | **H2** | **p-val** |
| All SNPs | Observed | 0.28 | 2.63E-02 | 0.25 | 6.21E-02 |
| All SNPs | 2% | 0.22 | 2.63E-02 | 0.19 | 6.21E-02 |
| All SNPs | 5% | 0.29 | 2.63E-02 | 0.25 | 6.21E-02 |
| All SNPs | 15% | 0.40 | 2.63E-02 | 0.35 | 6.21E-02 |
| All SNPs exclAPOE | Observed | 0.21 | 1.21E-01 | 0.15 | 2.53E-01 |
| All SNPs exclAPOE | 2% | 0.16 | 1.21E-01 | 0.12 | 2.53E-01 |
| All SNPs exclAPOE | 5% | 0.21 | 1.21E-01 | 0.15 | 2.53E-01 |
| All SNPs exclAPOE | 15% | 0.30 | 1.21E-01 | 0.22 | 2.53E-01 |
| All SNPs exclAPOE+GWAS | Observed | 0.19 | 1.48E-01 | 0.14 | 2.76E-01 |
| All SNPs exclAPOE+GWAS | 2% | 0.15 | 1.48E-01 | 0.11 | 2.76E-01 |
| All SNPs exclAPOE+GWAS | 5% | 0.19 | 1.48E-01 | 0.14 | 2.76E-01 |
| All SNPs exclAPOE+GWAS | 15% | 0.27 | 1.48E-01 | 0.20 | 2.76E-01 |
| Microglia | Observed | 0.26 | 1.63E-04 | 0.30 | 7.39E-05 |
| Microglia | 2% | 0.20 | 1.63E-04 | 0.23 | 7.39E-05 |
| Microglia | 5% | 0.26 | 1.63E-04 | 0.30 | 7.39E-05 |
| Microglia | 15% | 0.37 | 1.63E-04 | 0.43 | 7.39E-05 |
